# Trends in Incidence and Outcomes of Hospitalizations for Urinary Tract Infection among Older People in Spain (2001–2018)

**DOI:** 10.3390/jcm10112332

**Published:** 2021-05-26

**Authors:** Domingo Palacios-Ceña, Lidiane Lima Florencio, Valentín Hernández-Barrera, Cesar Fernandez-de-las-Peñas, Javier de Miguel-Diez, David Martínez-Hernández, David Carabantes-Alarcón, Rodrigo Jimenez-García, Ana Lopez-de-Andres, Marta Lopez-Herranz

**Affiliations:** 1 Department of Physical Therapy, Occupational Therapy, Rehabilitation and Physical Medicine, Universidad Rey Juan Carlos, Alcorcón, 28922 Madrid, Spain; domingo.palacios@urjc.es (D.P.-C.); lidiane.florencio@urjc.es (L.L.F.); cesar.fernandez@urjc.es (C.F.-d.-l.-P.).; 2 Preventive Medicine and Public Health Teaching and Research Unit, Health Sciences Faculty, Universidad Rey Juan Carlos, Alcorcón, 28922 Madrid, Spain; valentin.hernandez@urjc.es; 3 Respiratory Care Department, Hospital General Universitario Gregorio Marañón, Instituto de Investigación Sanitaria Gregorio Marañón (IiSGM), Universidad Complutense de Madrid, 28040 Madrid, Spain; javier.miguel@salud.madrid.org; 4 Department of Public Health & Maternal and Child Health, Faculty of Medicine, Universidad Complutense de Madrid, 28040 Madrid, Spain; dmartine@ucm.es (D.M.-H.); dcaraban@ucm.es (D.C.-A.); rodrijim@ucm.es (R.J.-G.); 5 Faculty of Nursing, Physiotherapy and Podology, Universidad Complutense de Madrid, 28040 Madrid, Spain; martal11@ucm.es

**Keywords:** urinary tract infections, aged, aged 80 and over, incidence, hospital mortality, epidemiologic studies

## Abstract

(1) Background: To assess time trends in the incidence and in-hospital outcomes of urinary tract infection (UTI) in older people (≥65 years) in Spain (2001–2018) according to sex and to identify the factors independently associated with in-hospital mortality (IHM). (2) Methods: Using the Spanish National Hospital Database, we analyzed comorbidity, procedures, diagnosis, isolated microorganisms, and outcomes. (3) Results: We included 583,693 admissions (56.76% women). In both sexes, the incidence increased with age over time (*p* < 0.001). In men and women, the highest IHM was found among the oldest patients (>84 years) with values of 9.16% and 8.6%, respectively. The prevalence of bacteremia increased from 4.61% to 4.98% from 2001 to 2018 (*p* < 0.001) and sepsis decreased from 3.16% to 1.69% (*p* < 0.001). The risk of dying increased with age (>84 years) (OR 3.63; 95% CI 3.5–3.78) and with almost all comorbidities. Urinary catheter was a risk factor for women (OR 1.1; 95% CI 1.02–1.18) and protective for men (OR 0.71; 95% CI 0.66–0.76). Isolation of *Staphylococcus aureus* increased the risk of IHM by 40% among men (OR 1.4; 95% CI 1.28–1.53). After adjusting for confounders, IHM decreased over time. (4) Conclusions: The incidence of UTIs was higher in older patients and increased over time; however, IHM decreased during the same period. Female sex, age, comorbidities, sepsis, and a *Staphylococcus aureus* isolation increased risk of IHM.

## 1. Introduction

Urinary tract infections (UTIs) are considered the third most common type of infections [1,2]. UTI incidence increases with age and is an important cause of antibiotic use, morbidity, and mortality in older adults [3]. Resistance to empirically prescribed antimicrobial agents is rising and this fact adds complications to the management of these infections among the elderly. UTIs in older women (>65) are seen at approximately double the rate of that in the female population overall [3]. Furthermore, it is estimated that, at a global level, after 65 years of age in noninstitutionalized people, the rate of UTI was 10.9% for men and 14% for women [4]. UTIs have been associated with several comorbidities such as vertebral fractures, rheumatic disease, multi-infarct dementia [4], undernourishment, diabetes, neuropathy, fecal and/or urinary incontinence [5,6,7], chronic diseases, and functional abnormalities [8,9,10]. Other factors predisposing to UTIs include estrogen deficiency and immune senescence [8], bed rest, hospitalizations, long-term medical institutionalization [5,6,7,8,10], iatrogenic factors, including anticholinergic agents, antibiotics, and the presence of urinary catheters [6,8].

The diagnosis of a UTI may be complicated among old patients [1] as classical symptoms and signs are frequently replaced by atypical symptoms, such as asymptomatic bacteriuria [6]. For example, bacteremia due to UTI in older patients may manifest as delirium instead of fever and chills [11]. In older patients, it is also difficult to apply the systemic inflammatory response syndrome criteria of temperature (>38 °C or <36 °C) used in sepsis diagnosis [11]. This increases the risk of underdiagnosing sepsis in older patients [6,11]. In addition, biomarkers used to diagnosis sepsis (ESR, CRP, lactate) may be elevated at baseline in older adults [11]. Moreover, comorbidities such as diabetes, chronic kidney disease, and pyelonephritis; age-related organ decline (decrease thyroid and endogenous corticosteroids hormones); malnutrition; and immunosenescence (decrease immune responses and cytokine production), increase the risk of severe infection, sepsis, and mortality in older adults [11,12].

More severe polymicrobial UTIs are more commonly found among older patients, who also suffer with a higher proportion multidrug-resistant bacterial infections [2,8]. In older adults, *E. coli* remains the most frequently isolated pathogen, as it is identified in more than two-thirds of cases [6,8,13,14]. 

In addition, UTIs result in a huge economic burdens to healthcare systems, leading to significant losses of income, morbidity, and restricted activity [1,3,5,10,15,16,17]. UTIs are responsible for one million emergency room visits in the USA and 100,000 hospitalizations each year [8]. Furthermore, in this country, among patients aged over 65 years, UTIs cause 15.5% of hospitalizations and 6.2% of deaths attributable to infectious disease [8]. UTIs, including cystitis and pyelonephritis, are among the most common infections in the outpatient setting [18]. The Global Prevalence Study on Infections in Urology (GPIU study) reported that, in Asia, the most common clinical diagnoses were pyelonephritis and cystitis, with a prevalence of 30.7% and 29.9%, respectively [13]. In the USA, risk factors for UTI-related hospitalization included severe dependency on activities of daily living, impaired decision making, history of UTI treatment, history of urinary catheter, and current urinary catheter [19].

In the UK and USA [5], the hospital admissions for UTIs in older people are increasing. 

To our knowledge, in Spain, no previous investigation to assess the incidence of hospitalizations among older people with a primary diagnosis of UTIs has been conducted. The objectives of the current study were: 1, to describe and analyze the incidence from 2001 to 2018 of UTIs hospitalizations according to sex, comorbidities and isolated pathogens, bacteremia, and sepsis among Spanish older people; 2, to assess time trends in hospital outcome variables such as in-hospital mortality (IHM), and 3, to identify the factors independently associated with IHM among older men and women.

## 2. Materials and Methods

### 2.1. Study Design and Data Collection

We conducted a retrospective observational study. Data were obtained from the Spanish National Hospital Discharge Database (SNHDD) for patients admitted to the hospital from year 2001 to 2018. Details of the database can be found elsewhere [20]. 

The International Classification of Diseases (ICD) was used for coding by the SNHDD, specifically the 9th Revision (ICD9) from 2001 to 2015 and the 10th Revision (ICD-10) from 2016 until now. 

We selected patients aged 65 year or over admitted to the hospital. Patients were identified as admissions with a diagnosis of UTI based on the definition of urinary tract infections of the Agency for Healthcare Research and Quality (AHRQ) [21,22]: according to the AHRQ, admissions with any principal diagnosis included in the list of ICD 9 and ICD 10 codes shown in Table S1 are considered urinary tract infections [21,22]. As can be seen in this table, these diagnoses include acute pyelonephritis, urethritis cystica, tubulo-interstitial nephritis, cystitis with or without hematuria, renal and perinephric abscess, renal tubulo-interstitial disease, pyelitis cystica, other urinary tract infection with site not specified, and pyeloureteritis cystica. The following diagnoses and conditions were excluded, as recommended by the AHRQ: kidney or urinary tract disorders, immunocompromised state admissions, and obstetric admissions [21,22].

Patients with only a code for asymptomatic bacteriuria (ICD 19 code 791.9 or ICD 10 code R82.71) without any of the codes specified in Table S1 as a primary diagnosis were not included as a urinary tract infection [21,22].

### 2.2. Study Variables 

The main study outcome variables were the incidence per 100,000 inhabitants of UTIs, LOHS, and IHM. The Charlson Comorbidity Index (CCI) was used to quantify comorbid conditions as described by Quan et al. [23]. The presence of isolated microorganisms, bacteremia, and sepsis was assessed using the ICD codes shown in Table S1. We specifically identified patients with codes for *Escherichia coli*, *Pseudomonas*, *Klebsiella pneumonia*, *Enterococcus*, *Proteus*, and *Staphylococcus aureus* in any diagnosis field. Finally, the variables “urinary catheter” and “urinary incontinence” were created using the procedure codes described in Table S2.

### 2.3. Statistical Analysis

To calculate the incidence rates, we divided the number of cases of UTIs in each sex and age group by the Spanish population in the corresponding period according to the Spanish National Statistical Institute [24]. For continuous variables, mean with standard deviation (SD) or median with interquartile range (IQR) are provided. Absolute numbers and proportions are shown for categorical variables

Age and sex adjusted Poisson regression models were used to assess time trends in incidences providing an incidence rate ratio (IRR) with 95% confidence intervals (CI). Time trends were analyzed using logistic regression, ANOVA, or a Kruskal–Wallis test for categorical variables, means, and medians, respectively. 

Multivariable logistic regression models for men, women, and both sexes were constructed to identify predictors of IHM among patients with UTIs. We included in the multivariable models all the independent variables with significant bivariate associations (*p* < 0.10) with the dependent variable and those considered scientifically relevant in other investigations. In order to fit the multivariable model, the importance of each variable was verified. This included an examination of the Wald statistic for each variable and a comparison of each estimated coefficient with the coefficient from the univariate model containing only that variable. Variables that did not contribute to the model based on these criteria were eliminated and a new model was fitted. The new model was compared to the previous model using the likelihood ratio test. Furthermore, estimated coefficients for the remaining variables were compared to those from the full model. This process of deleting, refitting, and verifying continued until all the important variables were included in the model. The “enter modeling” method of STATA 14.0 was used for this variable selection. Once the final model was obtained, collinearity between variables was assessed by the variance inflation factor, and interactions in the model analyzed.

The odds ratio (OR) and 95% confidence intervals (CI) were used to measure association.

As our investigation includes very large samples, we may find statistically significant results when the magnitude of these differences is small. To assess the relevance of the differences controlling for the sample size, we calculated the “effect sizes” using Cramer’s and Cohen’s method [25]. According to Cohen’s significance of associations, these are considered relevant if the effect size is over 0.2 [25]. In the tables, we marked those associations with an effect size of under 0.2.

### 2.4. Ethical Aspects

The SNHDD is provided freely to all investigators by the Spanish Ministry of Health. The characteristics of the database make it unnecessary to obtain approval by an ethics committee [20]. 

## 3. Results

The total number of hospital admissions in Spain from 2001 to 2018 with a principal diagnosis of UTI was 583,693. The proportion of women was 56.76% (331,275). Among men aged 65 years or over, the incidence per 100,000 inhabitants increased from 277.01 in 2001–2003 to 557.99 in 2016–2018 (*p* < 0.001). The equivalent figures for women were 256.86 and 556.96 (*p* < 0.001). Over the entire time period, the incidence was higher among men than women (417.28 vs. 407.4; *p* < 0.001).

Incidence rates per 100,000 for men and women from 2001 to 2018 according to age appear in Table 1. The incidence increased with age in all time periods for men, women, and both genders. The highest incidences among men and women were found among those aged > 84 years with incidence rates per 100,000 inhabitants of 1168.67 and 1062.12, respectively. Poisson regression analysis showed a significant increase from the period 2001–2003 to the period 2016–2018 in men and woman for all age groups studied (*p* < 0.001).

The clinical characteristics and hospital outcomes for admissions of elderly men with a principal diagnosis of UTI in Spain from 2001 to 2018 are shown in Table 2. The mean age rose two years over the study period (78.01 years to 79.99 years; *p* < 0.001). All the chronic conditions included in the CCI became significantly more prevalent in the period 2016–2018 when compared to the period 2001–2003, with the exception of hemiplegia or paraplegia and AIDs that remained unchanged and peptic ulcer that significantly decreased. However, as can be seen in the table, not all the trends for comorbidities showed an effect size over 0.2. The most prevalent conditions over the entire time period were diabetes (30.35%), chronic renal disease (20.57%), COPD (18.79%), dementia (12.11%), cancer (11.46%), and cerebrovascular disease (10.07%). The mean value of CCI increased from 1.08 to 1.49 over time (*p* < 0.001). As can be seen in Table 2, the proportion of patients with a urinary catheter was 5.57% in 2001–2003, rising to 8.51% in 2016–2018 (*p* < 0.001). For urinary incontinence, a change from 1.48% to 3.28% (*p* < 0.001) was found among men. Regarding hospital outcomes, the median LOHS remained unchanged in 6 days whereas the IHM decreased significantly from 6.46% to 5.15% from 2001–2003 to 2016–2018.

For women, the time trends in age, clinical characteristics, and hospital outcomes are shown in Table 3. Women have increased their mean age in more than three years over the study period from 79.7 years to 83.25 years (*p* < 0.001). The mean CCI rose significantly from 0.99 in 2001–2003 to 1.37 in 2016–2018. All the chronic conditions included in the CCI were more frequently found in the period 2016–2018 than in the period 2001–2003, with the exception of the same conditions described for men. As with men, not all the trends for comorbidities showed an effect size over 0.2. Among women, the most prevalent conditions found over the entire period were diabetes (25.19%), dementia (19,96%), chronic renal disease (17.68%), congestive heart failure (11.08%), and cerebrovascular disease (10.38%). The proportion of women with a urinary catheter increased from 1.98% to 3.83% over time (*p* < 0.001), the corresponding figures for urinary incontinence were 2.57% and 6.02%, respectively (*p* < 0.001). Median LOHS and the IHM decreased significantly (*p* < 0.001) among women from 7 days to 6 days and from 6.31% to 5.99%, respectively. When we compared between sexes, we found that men were younger, with higher mean CCI, more use of urinary catheter, and lower IHM (all comparisons *p* < 0.001).

**Figure 1:**
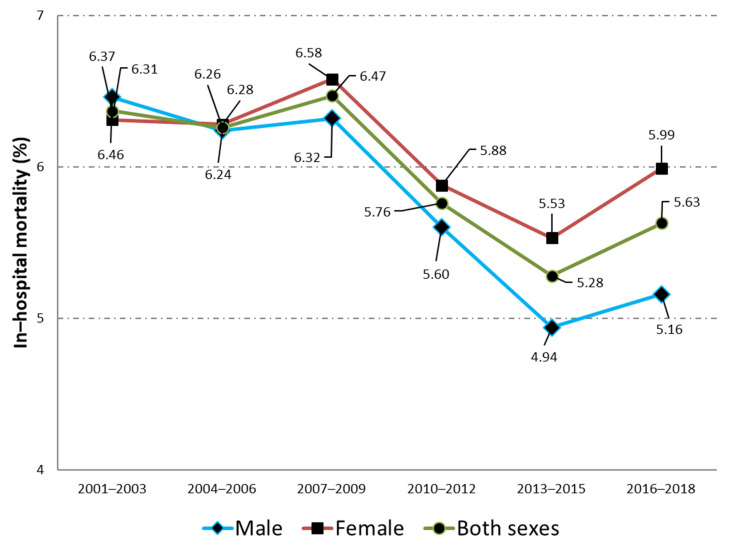
Time trend, from 2001–2003 to 201620–18, in the in-hospital mortality for men and women admitted to the hospital with a principal diagnosis of urinary tract infection.

Figure 1 shows the change over time in the IHM for men and women admitted to the hospital with a principal diagnosis of urinary tract infection (2001–2018). A detailed analysis of the time trend by age groups in the IHM is shown in Table 4. In men and women, the highest IHM was found among the oldest patients (>84 years) with values of 9.16% and 8.6%, respectively. For both sexes, a significant decrease over time was observed in all the age groups analyzed and for the total populations.

**Figure 2:**
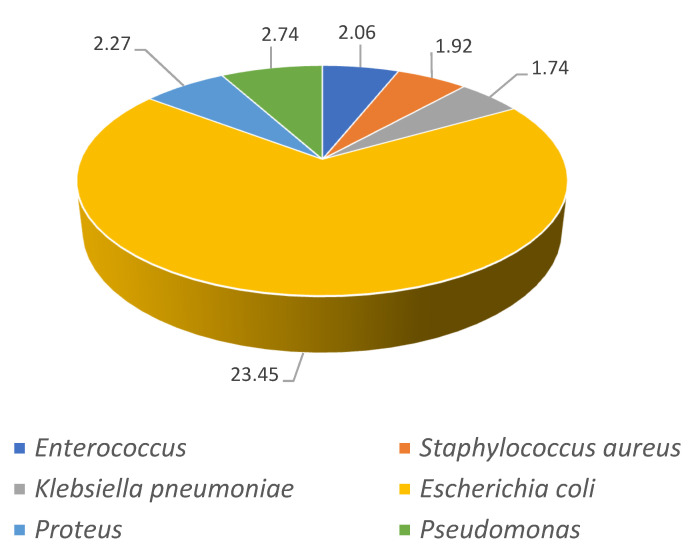
Prevalence of isolated pathogens for the first time period analyzed (2001–2003).

**Figure 3:**
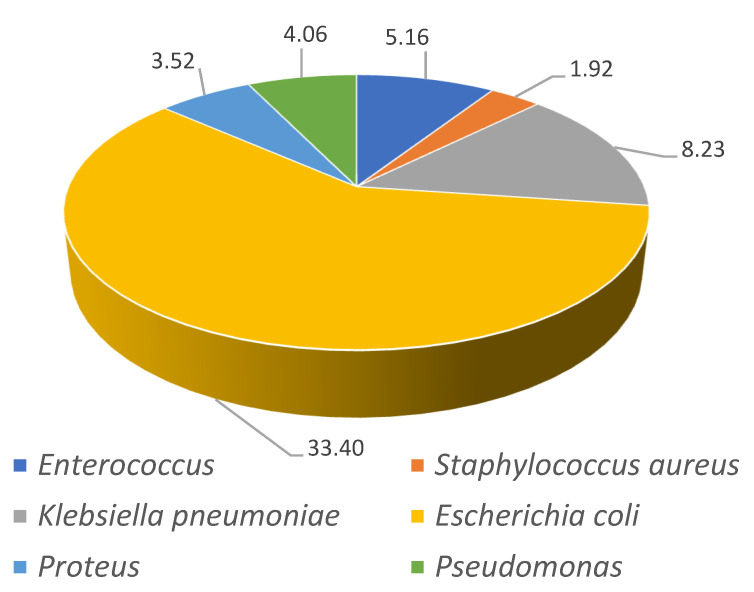
Prevalence of isolated pathogens for the last time period analyzed (2016–2018).

The isolated pathogens for the first and last time periods analyzed (2001–2003 and 2016–2018) are shown in Figure 2 and Figure 3. For both time periods, *Escherichia coli* was the most frequently diagnosed pathogen increasing 10% from 23% in 2001–2003 to 33% in 2016–2018 (*p* < 0.001). The increments in *Klebsiella pneumonia* from 1.74% to 8.23% (*p* < 0.001) and for Enterococcus (2.06% to 5.16%, *p* < 0.001) are very remarkable.

The prevalence of isolated pathogens, bacteremia, and sepsis among elderly men and women can be seen in Table 5. Among men, the most frequently isolated pathogens were *Escherichia coli* (25%), *Pseudomonas* (5.94%), *Klebsiella pneumonia* (5.24%), and *Enterococcus* (4.96%). The prevalence of all the pathogens analyzed increased significantly over time. Bacteremia was codified in 3.84% of hospitalized men in 2001–2003, increasing to 5.23% in 2016–2018 (*p* < 0.001), whereas sepsis decreased from 3.07% to 2% (*p* < 0.001). As can be seen in Table 5, the most prevalent pathogens isolated among women were *Escherichia coli* (34.55%), *Klebsiella pneumonia* (5.15%), *Enterococcus* (3.14%), and *Proteus* (2.96%). The prevalence of bacteremia increased from 4.61% to 4.98% from 2001–2003 to 2016–2018 (*p* < 0.001) and sepsis decreased from 3.16% to 1.69% (*p* < 0.001).

The variables associated with in-hospital mortality after multivariable analysis are shown in Table 6. For both sexes, the risk of dying increased with age and with suffering all the CCI conditions, except for diabetes, which had a protective effect, and COPD, rheumatoid disease, and peptic ulcer, which showed no association. Urinary catheter was a risk factor for IHM among women (OR 1.1; 95% CI 1.02–1.18) and showed the opposite, a protective effect, among men (OR 0.71; 95% CI 0.66–76). Regarding isolated pathogens, *Escherichia coli*, *Pseudomonas* (only men), *Klebsiella pneumonia*, *Enterococcus* (only women), and *Proteus* were associated to a lower risk of dying during hospitalization. Men with an isolation of *Staphylococcus aureus* had a 40% higher risk of IHM (OR1.4; 95% CI 1.28–1.53) compared with those without this pathogen. The equivalent OR among women was even higher (OR 1.6; 95% CI 1.46–1.76). Sepsis was a strong risk factor for dying among men (OR 5.41; 95% CI 4.91–5.97) and women (OR 6.5; 95% CI 5.97–7.09). Multivariable analysis confirmed the results of the bivariate analysis with a significant reduction in the IHM over time for men and women. Finally, women had a slightly but significantly higher probability of dying than men (OR 1.06; 95% CI 1.03–1.08).

## 4. Discussion

The current findings demonstrate a significant increase in UTI admissions incidence from 2001 to 2018 in both sexes. Moreover, the factors associated with IHM identified among elderly patients hospitalized due to a primary diagnosis of UTI were higher age, more comorbidities, *Staphylococcus aureus* isolation, and sepsis. However, women had significantly higher IHM than men. Nevertheless, there was a decrease in the IHM in elderly patients admitted for UTI between 2001 to 2018 in Spain.

The significant association between older age and higher incidence and IHM in patients with UTI observed here is supported by previous reports demonstrating that the risk of mortality increases in older ages [5,7,26,27,28]. It may be justified by the association between aging and a reduced innate and adaptive immune response, which is known as immune senescence [8]. It is mediated by T cell dysfunction and blunted cytokine-mediated inflammatory response. In addition, the higher risk of adverse outcomes for UTI in older patients may be due to an impairment of normal defense mechanisms such as acidification of the urine from organic acids, and immunoglobulin production [6,29].

Furthermore, there are differences in UTI-related IHM risk between women and men within the older population. Previous studies in hospital admissions of older chronic obstructive pulmonary disease (COPD) patients [27] and older patients with diabetes [7] with a principal diagnosis of UTI, reported that the oldest women (>84 years old) showed a higher risk for IHM when compared with the oldest men. COPD patients showed OR 18.27 (95% CI: 4.46–74.85) versus OR 5.33 (95% CI: 2.82–10.09), women versus men, respectively. In addition, women with diabetes showed OR 12.8 (95% CI: 8.2–19.97) versus the OR 9.8 (95% CI: 6.03–15.94) of men with diabetes [7].

The gender dimorphism in the morbidity profiles and mortality has already been signalized in hospitalized older people [30]. For women with a UTI, the clinical spectrum can vary from asymptomatic bacteriuria to symptomatic and recurrent infections, to sepsis associated with UTI requiring hospitalization [31,32]. This causes great challenges for diagnosis, management, and early treatment, together with antibiotics selected by identifying the urological pathogens, knowing local resistance rates, and considering adverse effect profiles. Moreover, the shorter urethral length of women frequent vaginal colonization, and the loss of pelvic floor muscle tone and the associated prolapse observed in older women make them more likely to UTIs [9,29]. 

We found that *Escherichia coli* was the most prevalent pathogen in both sexes regarding isolated pathogens, which is in line with previous reports [1,7,26,28,33,34,35]. However, the risk of IHM was associated with *Staphylococcus aureus*, but the risk decreased with other isolated pathogens, which is also observed among patients with COPD and type 2 diabetes mellitus [7,27]. *Staphylococcus aureus* bacteriuria is associated with bacteremia by this microorganism, which presents a complicated course with higher IHM [12,36,37,38].

The higher prevalence of comorbidities observed for the patients with UTI may also contribute to the clinical implications. Comorbidities not only influence UTI’s development [6] but also increases the risk of IHM. Among comorbidities that have been reported to increase the mortality risk are septic shock (OR 1.92, 95% CI: 0.93–3.98), metastatic cancer (OR 2.89, 95% CI: 1.46–5.73), and complicated UTI (OR 1.48, 95% CI: 1.04–2.11) [28], sepsis (OR 2.72, 95% CI: 1.50–4.94,), acute kidney injury with estimated glomerular filtration rate (eGFR) ≤ 29 (OR 6.50, 95% CI: 5.24–8.55) [5]. In Spain, the CCI was associated with mortality in hospital admissions of type 2 diabetes mellitus and COPD patients with a principal diagnosis of UTI, OR 2.44 (95% CI: 2.31–2.58) and OR 2.21 (95% CI: 2.15–2.28), respectively [7,27]. 

The mortality risk is also influenced by conditions related to the UTI. Eliakim-Raz et al. [28] showed that UTI admission (OR 5.07, 95% CI: 3.18–8.07), corticosteroid treatment (OR 1.92, 95% CI: 1.12–3.54), being bedridden (OR 2.11, 95% CI: 1.4–3.18), and complicated UTI (OR 1.48, 95% CI: 1.04–2.11) were prevalent conditions that contributed to substantially increasing UTI hospital mortality risk [37]. The risk of all-cause mortality within 60 days after the index UTI diagnosis is significantly higher with deferred antibiotics and no antibiotics than with immediate antibiotics (aHR 1.16, 95% CI: 1.06–1.27; aHR 2.18, 95% CI: 2.04–2.33, respectively). Moreover, Hsiao et al. [38] reported that very old patients (>80) with UTI, bacteremia (OR 2.54, 95% CI: 1.38–4.69), and acute kidney injury (OR 4.37, 95% CI: 2.15–8.90) were independently associated with uroseptic shock.

Nevertheless, our results showed that diabetes was associated significantly with decreased UTI risk mortality, although the opposite effect is usually expected. Similarly, de Miguel-Diez et al. [27] and López-de-Andrés et al. [7] reported an association between comorbidities and decreased hospital mortality risk, included type 2 diabetes mellitus for the total population (OR: 0.97, 95% CI: 0.91–1.01), for men (OR 0.95, 95% CI: 0.90–1.02), and women (OR 0.99, 95% CI: 0.93–1.04); and COPD among women admitted to the hospital with an episode of UTI (OR 0.99, 95% CI: 0.93–1.04). This association may, in part, be explained by: 1, greater awareness of UTIs and overall infection risk among older patients [29,39], which may be a consequence of the higher degree of vigilance among patients, family members, and healthcare providers to seek and report typical symptoms of UTI (dysuria, urinary frequency, fever, back/flank pain) and nonspecific symptoms (acute confusion, behavioral disturbances, falls); 2, more accessible outpatient health-care [29]; 3, early use of diagnostic techniques [39]; 4, higher hospitalization rate with less severe UTIs [9]; 5, better antibiotics, better hospital protocols for the treatment of infections [39]. Consequently, these would facilitate early detection, a greater likelihood of UTI diagnosis, and timely treatment.

We found that urinary catheter insertion was a risk factor of IHM among women; however, it had a protective effect in men. Similar results were reported in COPDs and diabetes patients with UTI [7,27]. Gomila et al. [40] reported no significant association between catheter-associated urinary tract infection and an increased risk of mortality (OR, 1.40; 95% CI: 0.77–2.54), despite being the most frequent cause of complicated UTI and affecting mainly frail patients. Controversially, Gyesi-Appiah et al. [41] showed that the presence of short-term indwelling urinary catheters increased the risk of infection, length of hospital stay, and mortality rate. These authors hypothesized that the association between a urinary catheter and IHM in women might be due to short-term urinary catheters’ uncontrolled excessive use [41]. This supports the advice to provide strict monitoring and to remove the short-term urinary catheters as soon as possible [41].

A significant decrease in mortality from 2001 to 2018 in both genders was identified. In recent studies, López-de-Andrés et al. [7] and de Miguel-Diez et al. [27] analyzed the in-hospital mortality of type 2 diabetes patients and COPD patients hospitalized with UTIs in Spain from 2001 to 2018. They found a reduction in all of them. This time trend reduction may be justified by improving diabetes and COPD control and UTI diagnosis and treatment over time.

Finally, current recommendations are that the treatment of these conditions should be adapted in the presence of complicated UTI, catheter-associated UTI, residential aged care setting, and presence of recurrent UTI [29]. Furthermore, preventive strategies should be encouraged in addition to antibiotic treatment [42]. 

Unfortunately, the Spanish National Hospital Discharge Database (SNHDD) does not include information on the antibiotic sensitivities of the organisms. In Spain like in other countries, antimicrobial resistance in common urinary pathogens is increasing at an alarming rate, as a result of the overuse and misuse of antibiotics [43,44,45]. The most recent Spanish paper reported that for *E. coli*, which continues to be the most frequent microorganism in UTIs, the sensitivity to amoxicillin, amoxicillin-clavulanic acid, trimethoprim-sulfamethoxazole, or quinolones has increased over the last years while the rate of sensitivity to fosfomycin and nitrofurantoin remained stable and above 95%. The non-sensitivities were higher in men and as age increased with 6% of *E. coli* identified as producers of extended-spectrum beta-lactamases [43,44,45].

The strengths of the current study include the large sample size and its representativeness considering the Spanish population. However, it also presents some limitations. First, a causal relationship cannot be established due to the study design. Second, no data on survival after hospital discharge are available. Third, ICD 9 and ICD 10 codes are susceptible to inaccurate registration or changes in the quality of coding over the years. However, the validity of ICD codes has been positively evaluated [46]. Fourth, the SNHDD does not include information on whether the patients admitted to the hospital were living at home with a catheter or if they were transferred to the hospital from a long-term care facility [20]. Finally, the case definition of our study may be affected by the lack of a standardized definition of the different types of UTI [4] and the wide spectrum of the disease, which makes the diagnosis of a UTI in elderly patients difficult.

## 5. Conclusions

The incidence of UTI hospitalizations increased from 2001 to 2018 among men and women and was higher as age rose in both sexes. On the other hand, the IHM decreased in both men and women during the same period. Higher IHM rates were associated with female sex, age, the presence of comorbidities (e.g., chronic liver disease, cancer, congestive heart failure, dementia), sepsis, and a *Staphylococcus aureus* isolation. However, even if the management of elderly with UTI seems to be improving in Spain, investigation and implementation of preventive programs is still necessary.

## Figures and Tables

**Table 1:** Hospital admissions among the elderly with a principal diagnosis of urinary tract infection per 100,000 inhabitants according to age groups and sex in Spain from 2001 to 2018.

		2001–2003	2004–2006	2007–2009	2010–2012	2013–2015	2016–2018	Total	*p*-Value
Sex	Age Groups	*n* (Inc/10^5^)	*n* (Inc/10^5^)	*n* (Inc/10^5^)	*n* (Inc/10^5^)	*n* (Inc/10^5^)	*n* (Inc/10^5^)	*n* (Inc/10^5^)	
MEN	65–74 years	8706 (161.54)	9572 (181.81)	10,854 (206.91)	11,789 (216.18)	15,102 (255.37)	18,328 (290.83)	74,351 (221.49)	<0.001
	75–84 years	10,541 (372.04)	13,318 (415.05)	17,018 (480.5)	19,883 (523.75)	22,354 (588.51)	25,255 (668.11)	108,369 (517.06)	<0.001
	>84 years	5312 (826.15)	6544 (929.91)	9395 (1084.28)	12,288 (1164.5)	15,414 (1234.06)	20,745 (1434.24)	69,698 (1168.67)	<0.001
	Total	24,559 (277.01)	29,434 (320.73)	37,267 (386.03)	43,960 (426.59)	52,870 (482.34)	64,328 (557.99)	252,418 (417.28)	<0.001
WOMEN	65–74 years	8769 (137.12)	9382 (151.39)	9405 (154.76)	10,364 (166.75)	11,859 (177.56)	13,438 (189.04)	63,217 (163.47)	<0.001
	75–84 years	13,278 (309.54)	16,813 (354.97)	20,626 (404.24)	24,852 (464.53)	27,408 (519.33)	29,841 (579.07)	132,818 (444.07)	<0.001
	>84 years	9235 (618.04)	11,859 (724.92)	18,002 (936.43)	24,457 (1084.81)	30,760 (1197.25)	40,927 (1432.6)	135,240 (1062.12)	<0.001
	Total	31,282 (256.86)	38,054 (302.75)	48,033 (366.61)	59,673 (431.79)	70,027 (482.09)	84,206 (556.96)	331,275 (407.4)	<0.001
BOTH	65–74 years	17,475 (148.29)	18,954 (165.36)	20,259 (178.92)	22,153 (189.85)	26,961 (214.1)	31,766 (236.87)	137,568 (190.43)	<0.001
	75–84 years	23,819 (334.4)	30,131 (379.23)	37,644 (435.48)	44,735 (489.11)	49,762 (548.28)	55,096 (616.75)	241,187 (474.14)	<0.001
	>84 years	14,547 (680.65)	18,403 (786.58)	27,397 (982.37)	36,745 (1110.22)	46,174 (1209.29)	61,672 (1433.15)	204,938 (1096.1)	<0.001
	Total	55,841 (265.35)	67,488 (310.33)	85,300 (374.85)	103,633 (429.57)	12,2897 (482.2)	148,534 (557.41)	583,693 (411.61)	<0.001

Inc/10^5^; Incidence per 100,000 inhabitants. *p* value for time trend using Poisson regression analysis adjusted by age and sex as appropriate.

**Table 2:** Clinical characteristics and hospital outcomes for admissions of elderly men with a principal diagnosis of urinary tract infection in Spain (2001–2018).

	2001–2003	2004–2006	2007–2009	2010–2012	2013–2015	2016–2018	Total	*p*-Value
Age; mean (SD)	78.01 (7.61)	78.54 (7.48)	79.15 (7.52)	79.56 (7.61)	79.63 (7.88)	79.99 (8.03)	79.35 (7.77)	<0.001
CCI; mean (SD)	1.08 (1)	1.2 (1.05)	1.28 (1.06)	1.37 (1.07)	1.4 (1.08)	1.49 (1.17)	1.35 (1.1)	<0.001
AMI; *n* (%)	984 (4.01)	1385 (4.71)	1768 (4.74)	1745 (3.97)	1949 (3.69)	3704 (5.76)	11,535 (4.57)	<0.001
CHF; *n* (%)	1199 (4.88)	1760 (5.98)	2530 (6.79)	3417 (7.77)	4215 (7.97)	6325 (9.83)	19,446 (7.7)	<0.001
PVD; *n* (%)	1059 (4.31)	1539 (5.23)	2119 (5.69)	2699 (6.14)	3453 (6.53)	4488 (6.98)	15,357 (6.08)	<0.001
CEVD; *n* (%) *	2297 (9.35)	2955 (10.04)	3899 (10.46)	4733 (10.77)	5416 (10.24)	6113 (9.5)	25,413 (10.07)	<0.001
Dementia; *n* (%)	2855 (11.63)	3312 (11.25)	4305 (11.55)	5214 (11.86)	5726 (10.83)	9162 (14.24)	30,574 (12.11)	<0.001
COPD; *n* (%) *	4471 (18.21)	5710 (19.4)	7007 (18.8)	8722 (19.84)	10,236 (19.36)	11,274 (17.53)	47,420 (18.79)	<0.001
Rheumatoid disease; *n* (%) *	186 (0.76)	286 (0.97)	405 (1.09)	644 (1.46)	777 (1.47)	885 (1.38)	3183 (1.26)	<0.001
Peptic ulcer; *n* (%) *	318 (1.29)	312 (1.06)	316 (0.85)	264 (0.6)	307 (0.58)	378 (0.59)	1895 (0.75)	<0.001
Diabetes; *n* (%)	5890 (22.53)	7695 (26.14)	10,733 (28.80)	13,783 (31.36)	19,959 (32.08)	21,905 (34.05)	76,608 (30.35)	<0.001
HP/PAPL; *n* (%)	336 (1.37)	395 (1.34)	511 (1.37)	644 (1.46)	815 (1.54)	901 (1.4)	3602 (1.43)	0.124
Chronic renal disease; *n* (%)	3071 (12.5)	4291 (14.58)	6495 (17.43)	9227 (20.99)	12,345 (23.35)	16,489 (25.63)	51,918 (20.57)	<0.001
Chronic liver disease; *n* (%) *	1055 (4.30)	1342 (4.54)	1697 (4.56)	2110 (4.80)	2554 (4.83)	3201 (4.98)	11,961 (4.74)	<0.001
Cancer; *n* (%)	2372 (9.66)	3264 (11.09)	4235 (11.36)	5186 (11.8)	6414 (12.13)	7463 (11.6)	28,934 (11.46)	<0.001
Metastatic cancer; *n* (%)	758 (3.09)	1138 (3.87)	1481 (3.97)	2011 (4.57)	2686 (5.08)	3484 (5.42)	11,558 (4.58)	<0.001
AIDS; *n* (%)	6 (0.02)	15 (0.05)	20 (0.05)	32 (0.07)	44 (0.08)	59 (0.09)	176 (0.07)	0.275
Urinary catheter; *n* (%)	1367 (5.57)	1887 (6.41)	3117 (8.36)	3953 (8.99)	5356 (10.13)	5477 (8.51)	21,157 (8.38)	<0.001
Urinary incontinence; *n* (%)	364 (1.48)	634 (2.15)	940 (2.52)	1516 (3.45)	1919 (3.63)	2112 (3.28)	7485 (2.97)	<0.001
LOHS; median (IQR)	6 (7)	6 (6)	6 (6)	6 (6)	6 (6)	6 (6)	6 (6)	0.089
IHM; *n* (%)	1586 (6.46)	1836 (6.24)	2356 (6.32)	2462 (5.6)	2614 (4.94)	3316 (5.15)	14,170 (5.61)	<0.001

CCI; Charlson Comorbidity index. AMI; acute myocardial infarction. CHF; congestive heart failure. PVD; peripheral vascular disease. CEVD; cerebrovascular disease. COPD; chronic obstructive pulmonary disease. HP/PAPL; hemiplegia or paraplegia. LOHS; length of hospital stay. IQR; interquartile range. IHM; in-hospital mortality. *p* value for time trend using ANOVA, logistic regression or Kruskall–Wallis test. * The effect size is under 0.2, so the association, even if significant, may not be relevant.

**Table 3:** Clinical characteristics and hospital outcomes for admissions of elderly women with a principal diagnosis of urinary tract infection in Spain (2001–2018).

	2001–2003	2004–2006	2007–2009	2010–2012	2013–2015	2016–2018	Total	*p*-Value
Age; mean (SD)	79.7 (7.78)	80.34 (7.65)	81.51 (7.58)	82.11 (7.64)	82.52 (7.72)	83.25 (7.8)	81.97 (7.79)	<0.001
CCI; mean (SD)	0.99 (0.92)	1.08 (0.96)	1.15 (0.98)	1.23 (1)	1.27 (1.02)	1.37 (1.08)	1.22 (1.02)	<0.001
AMI; *n* (%) *	605 (1.93)	913 (2.4)	1114 (2.32)	1138 (1.91)	1077 (1.54)	2190 (2.6)	7037 (2.12)	<0.001
CHF; *n* (%)	2293 (7.33)	3141 (8.25)	4606 (9.59)	6588 (11.04)	8224 (11.74)	11,858 (14.08)	36,710 (11.08)	<0.001
PVD; *n* (%) *	674 (2.15)	1053 (2.77)	1260 (2.62)	1545 (2.59)	1843 (2.63)	2109 (2.5)	8484 (2.56)	<0.001
CEVD; *n* (%)	2777 (8.88)	3726 (9.79)	5140 (10.7)	6708 (11.24)	7812 (11.16)	8219 (9.76)	34,382 (10.38)	<0.001
Dementia; *n* (%)	5645 (18.05)	6563 (17.25)	8809 (18.34)	11,122 (18.64)	12,724 (18.17)	21,264 (25.25)	66,127 (19.96)	<0.001
COPD; *n* (%)	1911 (6.11)	2508 (6.59)	3625 (7.55)	5201 (8.72)	6605 (9.43)	6890 (8.18)	26,740 (8.07)	<0.001
Rheumatoid disease; *n* (%)	661 (2.11)	955 (2.51)	1364 (2.84)	1988 (3.33)	2685 (3.83)	2851 (3.39)	10,504 (3.17)	<0.001
Peptic ulcer; *n* (%) *	247 (0.79)	246 (0.65)	237 (0.49)	257 (0.43)	253 (0.36)	319 (0.38)	1559 (0.47)	<0.001
Diabetes; *n* (%)	10,037 (32.08)	13,162 (34.59)	17,100 (35.60)	21,814 (36.55)	24,778 (35.38)	29,705 (35.27)	116,596 (25.19)	<0.001
HP/PAPL; *n* (%)	236 (0.75)	273 (0.72)	330 (0.69)	504 (0.84)	596 (0.85)	658 (0.78)	2597 (0.78)	0.071
Chronic renal disease; *n* (%)	3010 (9.62)	4437 (11.66)	6563 (13.66)	10,280 (17.23)	14,370 (20.52)	19,913 (23.65)	58,573 (17.68)	<0.001
Chronic liver disease; *n* (%) *	1306 (4.18)	1849 (4.86)	2241 (4.67)	2848 (4.76)	3351 (4.78)	3794 (4.50)	15,389 (4.64)	<0.001
Cancer; *n* (%)	1065 (3.4)	1454 (3.82)	1946 (4.05)	2506 (4.2)	3141 (4.49)	3781 (4.49)	13,893 (4.19)	<0.001
Metastatic cancer; *n* (%) *	464 (1.48)	627 (1.65)	870 (1.81)	1170 (1.96)	1488 (2.12)	1879 (2.23)	6498 (1.96)	<0.001
AIDS; *n* (%)	3 (0.01)	6 (0.02)	7 (0.01)	7 (0.01)	14 (0.02)	21 (0.02)	58 (0.02)	0.362
Urinary catheter; *n* (%)	619 (1.98)	1025 (2.69)	1670 (3.48)	2467 (4.13)	3386 (4.84)	3224 (3.83)	12,391 (3.74)	<0.001
Urinary incontinence; *n* (%)	805 (2.57)	1345 (3.53)	2266 (4.72)	3305 (5.54)	4596 (6.56)	5065 (6.02)	17,382 (5.25)	<0.001
LOHS; median (IQR)	7 (7)	7 (7)	6 (7)	6 (6)	6 (6)	6 (6)	6 (6)	<0.001
IHM; *n* (%)	1973 (6.31)	2391 (6.28)	3161 (6.58)	3511 (5.88)	3874 (5.53)	5043 (5.99)	19,953 (6.02)	<0.001

CCI; Charlson Comorbidity index. AMI; acute myocardial infarction. CHF; congestive heart failure. PVD; peripheral vascular disease. CEVD; cerebrovascular disease. COPD; chronic obstructive pulmonary disease. HP/PAPL; hemiplegia or paraplegia. LOHS; length of hospital stay. IQR; interquartile range. IHM; in-hospital mortality. *p* value for time trend using ANOVA, logistic regression, or Kruskall–Wallis test. * The effect size is under 0.2, so the association, even if significant, may not be relevant.

**Table 4:** Trends in the in-hospital mortality among elderly patients admitted to hospital with a principal diagnosis of urinary tract infection according to age groups and sex in Spain (2001–2018).

		2001–2003	2004–2006	2007–2009	2010–2012	2013–2015	2016–2018	Total	*p*-Value
Sex	Age Groups	IHM *n* (%)	IHM *n* (%)	IHM *n* (%)	IHM *n* (%)	IHM *n* (%)	IHM *n* (%)	IHM *n* (%)	
MEN	65–74 years	308 (3.54)	304 (3.18)	357 (3.29)	328 (2.78)	337 (2.23)	445 (2.43)	2079 (2.8)	<0.001
	75–84 years	682 (6.47)	878 (6.59)	1014 (5.96)	1042 (5.24)	995 (4.45)	1099 (4.35)	5710 (5.27)	<0.001
	>84 years	596 (11.22)	654 (9.99)	985 (10.48)	1092 (8.89)	1282 (8.32)	1772 (8.54)	6381 (9.16)	<0.001
WOMEN	65–74 years	258 (2.94)	272 (2.9)	278 (2.96)	244 (2.35)	265 (2.23)	317 (2.36)	1634 (2.58)	<0.001
	75–84 years	800 (6.03)	981 (5.83)	1163 (5.64)	1212 (4.88)	1205 (4.4)	1330 (4.46)	6691 (5.04)	<0.001
	>84 years	915 (9.91)	1138 (9.6)	1720 (9.55)	2055 (8.4)	2404 (7.82)	3396 (8.3)	11,628 (8.6)	<0.001
BOTH	65–74 years	566 (3.24)	576 (3.04)	635 (3.13)	572 (2.58)	602 (2.23)	762 (2.4)	3713 (2.7)	<0.001
	75–84 years	1482 (6.22)	1859 (6.17)	2177 (5.78)	2254 (5.04)	2200 (4.42)	2429 (4.41)	12,401 (5.14)	<0.001
	>84 years	1511 (10.39)	1792 (9.74)	2705 (9.87)	3147 (8.56)	3686 (7.98)	5168 (8.38)	18,009 (8.79)	<0.001
	Total	3559 (6.37)	4227 (6.26)	5517 (6.47)	5973 (5.76)	6488 (5.28)	8359 (5.63)	34,123 (5.85)	<0.001

Inc/10^5^ Incidence per 10 < 0.001 inhabitants. *p* value for time trend using logistic regression analysis.

**Table 5:** Isolated pathogens, bacteremia, and sepsis codified in hospital admissions among elderly patients with a principal diagnosis of urinary tract infection according to sex in Spain (2001–2018).

	Isolated Pathogen	2001–2003	2004–2006	2007–2009	2010–2012	2013–2015	2016–2018	Total	*p*-Value
**MEN**	*Escherichia coli n* (%)	4548 (18.52)	6217 (21.12)	8524 (22.87)	11,571 (26.32)	14,492 (27.41)	17,752 (27.6)	63,104 (25)	<0.001
	*Pseudomonasn* (%)	1005 (4.09)	1409 (4.79)	2043 (5.48)	2833 (6.44)	3654 (6.91)	4044 (6.29)	14,988 (5.94)	<0.001
	*Klebsiella pneumoniae n* (%)	429 (1.75)	667 (2.27)	1163 (3.12)	1937 (4.41)	3582 (6.78)	5441 (8.46)	13,219 (5.24)	<0.001
	*Enterococcu*s *n* (%)	672 (2.74)	944 (3.21)	1438 (3.86)	2074 (4.72)	3232 (6.11)	4154 (6.46)	12,514 (4.96)	<0.001
	*Proteus n* (%)	544 (2.22)	715 (2.43)	1040 (2.79)	1305 (2.97)	1810 (3.42)	2419 (3.76)	7833 (3.1)	<0.001
	*Staphylococcus aureus n* (%)	588 (2.39)	832 (2.83)	1116 (2.99)	1245 (2.83)	1438 (2.72)	1698 (2.64)	6917 (2.74)	<0.001
	Bacteriemia *n* (%)	944 (3.84)	1307 (4.44)	1890 (5.07)	2497 (5.68)	3060 (5.79)	3362 (5.23)	13,060 (5.17)	<0.001
	Sepsis *n* (%)	753 (3.07)	839 (2.85)	1067 (2.86)	1156 (2.63)	1091 (2.06)	1286 (2)	6192 (2.45)	<0.001
**WOMEN**	*Escherichia coli n* (%)	8547 (27.32)	11,438 (30.06)	15,215 (31.68)	21,026 (35.24)	26,256 (37.49)	31,969 (37.97)	114,451 (34.55)	<0.001
	*Pseudomonas n* (%)	524 (1.68)	749 (1.97)	1101 (2.29)	1479 (2.48)	1844 (2.63)	1991 (2.36)	7688 (2.32)	<0.001
	*Klebsiella pneumoniae n* (%)	541 (1.73)	957 (2.51)	1570 (3.27)	2729 (4.57)	4495 (6.42)	6778 (8.05)	17,070 (5.15)	<0.001
	*Enterococcus n* (%)	478 (1.53)	758 (1.99)	1232 (2.56)	1848 (3.1)	2587 (3.69)	3511 (4.17)	10,414 (3.14)	<0.001
	*Proteus n* (%)	725 (2.32)	931 (2.45)	1326 (2.76)	1749 (2.93)	2267 (3.24)	2808 (3.33)	9806 (2.96)	<0.001
	*Staphylococcus aureus n* (%)	485 (1.55)	654 (1.72)	934 (1.94)	1007 (1.69)	1177 (1.68)	1148 (1.36)	5405 (1.63)	<0.001
	Bacteriemia *n* (%)	1443 (4.61)	2052 (5.39)	2538 (5.28)	3297 (5.53)	3978 (5.68)	4195 (4.98)	17,503 (5.28)	<0.001
	Sepsis *n* (%)	989 (3.16)	1100 (2.89)	1266 (2.64)	1438 (2.41)	1269 (1.81)	1423 (1.69)	7485 (2.26)	<0.001

**Table 6:** Variables associated with in-hospital mortality in hospital admissions of elderly patients with a principal diagnosis of urinary tract infection according to sex in Spain (2001–2018).

	MEN	WOMEN	BOTH
	OR (95% CI)	OR (95% CI)	OR (95% CI)
75–84 years	1.89 (1.79–1.99)	2.04 (1.92–2.16)	1.96 (1.89–2.04)
>84 years	3.6 (3.41–3.8)	3.71 (3.51–3.92)	3.63 (3.5–3.78)
AMI	1.17 (1.08–1.26)	1.35 (1.24–1.48)	1.24 (1.17–1.32)
CHF	2.03 (1.93–2.14)	1.79 (1.72–1.86)	1.88 (1.82–1.94)
PVD	1.15 (1.08–1.24)	1.52 (1.41–1.64)	1.3 (1.23–1.37)
CEVD	1.37 (1.3–1.44)	1.32 (1.27–1.38)	1.35 (1.3–1.39)
Dementia	1.67 (1.59–1.75)	1.42 (1.37–1.47)	1.5 (1.46–1.55)
Diabetes	0.95 (0.91–0.99)	0.97 (0.93–1)	0.95 (0.93–0.98)
HP/PAPL	1.65 (1.43–1.9)	1.97 (1.71–2.27)	1.8 (1.62–1.99)
Chronic renal disease	1.27 (1.21–1.32)	1.26 (1.21–1.31)	1.27 (1.23–1.3)
Chronic liver disease	2.09 (1.78–2.45)	1.8 (1.55–2.09)	1.93 (1.73–2.16)
Cancer	1.49 (1.42–1.57)	1.73 (1.62–1.84)	1.57 (1.51–1.64)
Metastatic cancer	4.04 (3.8–4.3)	3.85 (3.56–4.17)	3.94 (3.76–4.14)
Urinary catheter	0.71 (0.66–0.76)	1.1 (1.02–1.18)	0.87 (0.83–0.91)
*Escherichia coli*	0.51 (0.49–0.54)	0.45 (0.44–0.47)	0.47 (0.46–0.49)
*Pseudomonas*	0.72 (0.67–0.78)	NS	0.82 (0.77–0.87)
*Klebsiella pneumoniae*	0.71 (0.65–0.78)	0.75 (0.7–0.8)	0.73 (0.69–0.77)
*Enterococcus*	NS	0.92 (0.84–0.99)	0.92 (0.87–0.98)
*Proteus*	0.73 (0.66–0.81)	0.83 (0.76–0.9)	0.79 (0.74–0.84)
*Staphylococcus aureus*	1.4 (1.28–1.53)	1.6 (1.46–1.76)	1.49 (1.4–1.59)
Sepsis	5.41 (4.91–5.97)	6.5 (5.97–7.09)	5.98 (5.61–6.38)
Time periods (Continous)	0.97 (0.96–0.98)	0.98 (0.97–0.99)	0.98 (0.97–0.99)
Women	NA	NA	1.06 (1.03–1.08)

AMI; acute myocardial infarction. CHF; congestive heart failure. PVD; peripheral vascular disease. CEVD; cerebrovascular disease. COPD; chronic obstructive pulmonary disease. HP/PAPL; hemiplegia or paraplegia. NS; not significant. NA; not available. The reference categories for age is “65–74 years”, for sex “men”, and for the remaing variables, the reference category was the absence of the variable listed (for example “not AMI”).

## Data Availability

Any investigator can apply for the databases used in this investigation at https://www.mscbs.gob.es/estadEstudios/estadisticas/estadisticas/estMinisterio/SolicitudCMBDdocs/2018_Formulario_Peticion_Datos_RAE_CMBD.pdf.

## Supplementary Materials

The following are available online at https://www.mdpi.com/article/10.3390/jcm10112332/s1, Table S1: ICD 9 and ICD 10 diagnosis codes for urinary tract infection according to the Agency for Healthcare Research and Quality. Table S2: ICD 9 and ICD 10 codes used to identify pathogen isolations and conditions included in this investigation.
