# Supplementary material for: Trends in Incidence and Outcomes of Hospitalizations for Urinary Tract Infection among Older People in Spain (2001–2018)
Source: J Clin Med. 2021 May 26;10(11):2332. doi: 10.3390/jcm10112332 (PMC8198653; doi:10.3390/jcm10112332)
Supplement: Supplementary file 1 [file jcm-10-02332-s001.zip › jcm-1201752-supplementary.pdf]

Table S1. ICD 9 and ICD 10 diagnosis codes for Urinary Tract Infection according to the Agency for Healthcare Research and Quality

Diagnosis	ICD 9 codes	ICD 10 codes
Acute pyelonephritis	590.10, 590.11	N10
Urethritis cystica	590.3	N28.86
Tubulo-Interstitial nephritis, not specified as acute or chronic	590.80	N12
Acute cystitis without hematuria	595.0	N30.00
Renal and perinephric abscess	590.2	N15.1
Acute cystitis with hematuria	595.0	N30.01
Renal tubulo-interstitial disease, unspecified	590.9	N15.9
Cystitis, unspecified without hematuria	595.9	N30.90
Renal tubulo-interstitial disorders in diseases classified elsewhere	590.81	N16
Cystitis, unspecified with hematuria	595.9	N30.91
Pyelitis cystica	590.3	N28.84
Urinary tract infection, site not specified	599.0	N39.0
Pyeloureteritis cystica	590.3	N2885

Table S2. ICD 9 and ICD 10 codes used to identify pathogen isolations and conditions included in this investigation.

DIAGNOSIS/PROCEDURES	ICD 9 CM	ICD 10 CM
Escherichia coli	041.4; 041.41; 041.42; 041.43; 041.49; 038.42	B96.21; B96.22; B96.23; B96.20 B96.29; A41.51
Pseudomonas (aeruginosa) (mallei) (pseudomallei)	041.7; 038.43	B96.5; A41.52
Klebsiella pneumoniae	041.3	B96.1
Enterococcus	041.04	B95.2; A40.2; A41.81
Proteus (mirabilis) (morganii)	041.6	B96.4
Staphylococcus aureus. (methicillin susceptible) (methicillin resistant)	041.1; 041.10; 041.11; 041.12; 041.19; 038.1; 038.10; 038.11; 038.12; 038.19	B95.6; B95.61; B95.62; B95.7; B95.8; A41.0; A41.1; A41.2 A41.01; A41.02
Bacteriemia	790.7	R78.81
Sepsis	038.0; 038.1: 038.10; 038.11; 038.12; 038.19; 038.2; 038.3; 038.4; 038.40; 38.41; 038.42; 038.43; 038.44; 038.49; 038.8; 038.9	A40.0; A40.1; A40.l; A40.3; A40.8; A40.9; A41; A41.0; A41.01; A41.02; 41.1;41.2; A41.3; A41.4; A41.5; A41.50; A41.51; A41.52; A41.53; A41.59; A41.8; A41.81; A41.89; A41.9
Urinary catheter	57.94; 57.95	0T9B70Z; 0T9B80Z; 0T2BX0Z
Urinary incontinence	788.3x, 788.91	R32, N39.3, N39.4XX, R39.81
